# Effects of Elemental Mercury Vapor Inhalation on Arterial Blood Gases, Lung Histology, and Interleukin-1 Expression in Pulmonary Tissues of Rats

**DOI:** 10.1155/2021/4141383

**Published:** 2021-09-29

**Authors:** Liqaa A. Raffee, Khaled Z. Alawneh, Ruba A. Alassaf, Abdallah Alzoubi, Musa A. Alshehabat, Nadeem Alabdallah, Abdel-Hameed Al-Mistarehi

**Affiliations:** ^1^Department of Accident and Emergency Medicine, Faculty of Medicine, Jordan University of Science and Technology, Irbid, Jordan; ^2^Department of Diagnostic Radiology and Nuclear Medicine, Faculty of Medicine, Jordan University of Science and Technology, Irbid, Jordan; ^3^Department of Legal Medicine, Toxicology and Forensic Medicine, Faculty of Medicine, Jordan University of Science and Technology, Irbid, Jordan; ^4^Department of Pharmacology, Faculty of Medicine, Jordan University of Science and Technology, Irbid, Jordan; ^5^Department of Clinical Veterinary Medical Sciences, Faculty of Veterinary Medicine, Jordan University of Science and Technology, Irbid, Jordan; ^6^Faculty of Medicine, Jordan University of Science and Technology, Irbid, Jordan; ^7^Department of Public Health and Family Medicine, Faculty of Medicine, Jordan University of Science and Technology, Irbid, Jordan

## Abstract

We investigated the effects of elemental mercury vapor inhalation on arterial blood gases (ABGs), lung histology, and interleukin-1 (IL-1) expression in pulmonary tissues in rats. A total of 42 Sprague Dawley rats were divided randomly into three groups. Rats in the first group were used as the control (CG). A short-term group (STG) and a long-term group (LTG) were exposed to 500 *μ*g/m^3^ of mercury vapor 2 hrs/day for 21 days and 65 days, respectively. After exposure periods were completed, arterial blood samples were obtained, and ABGs were measured. Lung tissue sections were prepared for histology evaluation and immune-stained to detect IL-1 expression. There was a significant decrease in body weight in both STG (15%) and LTG (22%) compared with the CG. In the LTG, six out of 14 (43%) rats died, including two males and four females, while none of the rats in the STG died during the experiment. In both STG and LTG, a significant acid-base imbalance was characterized by a significant decrease in blood pH values and a significant increase in PCO_2_ values. Both PO_2_ and SpO_2_ blood values were significantly decreased in the STG and LTG, while no changes were observed in HCO_3_ values in all groups. Histological evaluation of lung tissues revealed severe lesions characterized by pulmonary emphysema and inflammatory cellular infiltrate. IL-1 expression in lung tissues was not significantly different between exposed rats and control subjects. These results indicate significant alterations in blood acid-base status characterized by severe respiratory acidosis with hypoxemia and no evidence of compensatory alkalosis in rats after exposure to short- and long-term elementary mercury vapor.

## 1. Introduction

Mercury is a highly toxic heavy metal with significant public health and safety implications worldwide [1–5]. Mercury is commonly found in nature in many different forms [4, 6, 7]. Inorganic mercury includes metallic mercury, mercury vapor (Hg^0^), and mercurous (Hg^+2^) or mercuric salts, while the organic form of mercury includes compounds containing carbon atoms such as methyl, ethyl, or phenyl groups [8]. All forms of mercury compounds mentioned earlier can be found, and chemically interchangeable, in the environment [9].

According to the World Health Organization (WHO), most human exposure to mercury occurs through the inhalation of elemental mercury vapor via occupational or dental amalgam exposure [7, 10]. The ingestion of seafood contaminated with organic mercury has been reported as another significant route to expose people to mercury [7]. Although mercury is present naturally in the environment, recent human industrial activities have resulted in the dangerous accumulation of more mercury in the land, water, and food supplies [7, 11]. This accumulation of mercury in the environment carries grave health risks and consequences. The most infamous case of mercury poisoning of the 20^th^ century is the “Minamata disease” incident, with the first cases noted in 1956 [12]. Industrial waste containing methylmercury was being released into Minamata Bay in Japan by a Japanese chemical factory, thus reaching the locals through contaminated fish as food. Thousands of locals have been affected since then, and even babies born in the 1960s–1970s to mothers who have been exposed to the contaminated fish were noted to have brain damage, mental retardation, and a variety of other diseases [9]. Industrial regulations have been put into action ever since recognizing the toxic nature of mercury and its ability to cross the blood-brain barrier. However, industrial activities such as coal combustion still produce high mercury levels disposed of in the atmosphere, land, and water [13].

The pathogenesis of mercury poisoning is often multifaceted as it manifests in many forms and can impact all body systems depending on the underlying pathways and enzymes affected. This vast pathogenic potential is due to the tendency of mercury to bind to sulfur groups [9], which are an essential component of the chemical structure of cellular proteins, enzymes, channels, and pumps, thereby disrupting their physiological function and inducing pathological change. One molecular effect of mercury is inhibiting vascular endothelial enzymes such as Na/K-ATPase and Ca^2^-ATPase, leading to disrupted vascular reactivity [14]. Another effect that mercury has on the body vasculature is mercury-induced nitric oxide (NO) inhibition, as a result of endothelial nitric oxide synthase (NOS) pathway inhibition [15]. This leads to the disruption of normal vasodilation and vasoconstriction of the vasculature. Mercury has also been shown to increase the production of reactive oxygen species (ROS), which in turn led to the inactivation of numerous enzymes such as paraoxonase, glutathione peroxidase, phospholipase D, and mitogen-activated protein kinases (MAPKs) [2, 14, 16]. MAPKs are extremely important to the functioning of the immune system, as they play an essential role in T-cell activation. Haase et al. (2010) demonstrated that mercury binding to MAPKs did not significantly result in the dysfunction of MAPKs. However, the overproduction of ROS triggered by mercury was the cause of the MAPKs dysfunction [16]. Mercury has also been associated with developing clinical manifestations of metabolic syndrome such as obesity, insulin resistance, and hypertension [17]. Tinkov et al. (2015) proposed that mercury affects the renin-angiotensin-aldosterone system (RAAS), leading to hypertension and altering *β*-cell functionality leading to insulin resistance [17].

The interleukin-1 (IL-1) cytokines act to regulate proinflammatory mediators in tissue injury [18]. The effect of mercury on IL-1 expression has been demonstrated in some studies, but the results have been inconclusive [19, 20]. IL-1 production has been shown to increase due to the presence of mercury in the tissue [19], while in another study, mercury was shown to have the opposite effect and reduce IL-1 expression [20].

Elemental mercury vapor inhalation has been shown to lead to direct lung tissue injury, capillary destruction, pulmonary edema, and eventually fibrosis [21]. Acute severe exposure to elemental mercury vapor has been reported to lead to fatality as a result of pulmonary insufficiency and acute renal failure [21]. Generally, mercury exposure is chronic at a low dosage, resulting in subtle toxic manifestations characterized by loss of appetite, weakness, malaise, loss of weight, and gastrointestinal upset [4, 7]. However, more severe manifestations of the immune system, gastrointestinal tract, renal, cardiopulmonary, and nervous systems have been reported in the acute form [4, 22]. Rapid recognition of mercury poisoning and its complications is critical in avoiding poor patient outcomes and lifesaving. The clinical management of mercury vapor poisoning revolves around maintaining ventilation, decontamination, chelation, and treating complications [23]. In severe acute cases with high plasma concentrations of mercury, plasma exchange can also be used [24].

To our knowledge, no recent scientific reports are documenting the effects of exposure to elementary mercury vapor on various blood gas parameters and underlying pulmonary lesions that might explain possible acid-base alterations associated with inhalation of mercury vapor. Therefore, this study was designed to investigate the toxic effects of elemental mercury vapor on various arterial blood gas parameters and determine the possible underlying pulmonary pathology that might lead to acid-base alterations using Sprague Dawley rats. A preliminary preprint version of this scientific article was published before peer review on the Research Square website [25].

## 2. Materials and Methods

### 2.1. Animals

All experimental procedures performed in this study were reviewed and approved by the Institutional Animal Care and Use Committee (IACUC) of the Jordan University of Science and Technology (JUST). A total of 42 adult Sprague Dawley rats weighing between 150 and 200 grams were used in the study. Rats were randomly divided into three equal groups; 14 rats for each group with seven males and seven females. The first group received no mercury vapor exposure and was defined as a control group (CG). In the second group (short-term group, STG) and the third group (long-term group, LTG), rats were exposed to 500 *μ*g/m^3^ of elementary mercury vapor for 2 hours daily for 21 days and 65 days, respectively. This used concentration of elementary mercury vapor was chosen based on the available animal studies of the lowest observed adverse effects dose and lethal dose for subchronic and chronic mercury inhalation exposures [1, 26–28]. Exposure was limited to 2-hour duration per day to minimize the stress caused by restraint. Rats were housed individually in cages during the experiment and offered feed and fresh drinking water ad libitum. They were housed in a temperature- and humidity-controlled room with high-efficiency particulate air (HEPA) filtered air to keep the room pathogen-free throughout the experiment. The room temperature was maintained at 22–25°C with day/night cycles of 12/12 hours.

### 2.2. Experimental Design

In this experiment, STG and LTG rats were exposed to elemental mercury vapor (Shijiazhuang Shuliang Commerce Trade Co., China) using a stainless steel exposure chamber (50 cm × 50 cm × 80 cm) with plastic top cover and rubber sealing as described previously [1]. The chamber on its top was connected by a tube to an oxygen cylinder to provide pure oxygen at 10 liters/min rate during the mercury vapor exposure. The chamber was also connected to a vacuum pump on its lower part to suck out the air saturated with mercury vapor at the end of exposure and provide the exposure chamber with fresh air. Chamber temperatures were maintained at 22–24°C. Metallic mercury was injected at 7.3 *μ*l using a micropipette and heated in a small glass bottle adjacent to the exposure chamber. The mercury vapor exposure was generated by passing heated air over the mercury and then mixed with the primary air stream flowing into the exposure chamber. The resulting mercury vapor concentration was controlled by adjusting the flow of heated air and was monitored continuously during exposure with an ultraviolet mercury monitor. The monitor's calibration was confirmed using a Jerome 431-X mercury vapor analyzer (Arizona Instrument LLC, Chandler, AZ).

The 2-hour session was designated as the mercury vapor value reached 66% of the target concentration within approximately 10 minutes, and it reached 90% of the target value within an additional 1-2 min. Once achieved, the concentration was maintained at the target level of 500 *μ*g/m^3^ (±5%). At the end of the experiment, the mercury vapor generator was turned off, and the airflow was stopped. The chamber concentration declined rapidly, falling to ≤30 *μ*g/m^3^ in a few minutes. The rats were then removed after another 30 minutes in order to exhaust the chambers completely. This procedure was repeated for 21 days for the STG and 65 days for the LTG.

All animals were treated humanely with the alleviation of suffering. All were monitored daily by the research and personnel staff from JUST. All personnel and researchers were responsible for strictly experimenting, following the safety protocols and procedures, and adhering to the university laboratory policies to ensure their safety. The research team wore personal protective equipment (PPE) during the experiment, including gloves, head caps, shoe covers, gowns, and N95 face masks. Micropipette cover, mercury residuals, and PPE used in the experiment were kept in plastic zipper bags inside a biohazard box and then handed over to the safety, occupational, and environmental health department at JUST.

### 2.3. Clinical Monitoring

Rats were closely monitored for abnormal signs such as respiratory distress, weakness, or stool changes during the exposure and afterward.

### 2.4. Arterial Blood Sample Collection

After completing the exposure periods (21 days for the STG and 65 days for the LTG), arterial blood was collected from each rat under light sedation using ether in a glass chamber. Arterial blood was collected via cardiac puncture of the left ventricle using heparinized syringes attached to 22 gauge needles (Becton, Dickinson, and Company, USA). Arterial blood gases (ABGs) were measured immediately using a blood gas analyzer (Cobas; Roche Diagnostics, Switzerland).

### 2.5. Necropsy, Histopathology, and Lung Injury Estimation

After arterial blood was collected, rats were humanely euthanized using ether overdose in a glass chamber. A thorough necropsy was performed on all rats, and any abnormal gross findings were recorded. Tissue samples from both lungs were collected and placed immediately in 10% neutral buffered formalin. A portion of the tissue samples was processed, stained with H&E, and imaged using a digital light microscope for manual histopathology analysis as described previously [29]. Light microscopy of the lung could provide qualitative evidence supportive of lung injury and hydrostatic edema, such as thickening of alveolar septa, alveolar flooding, and perivascular or airway cuffing. Also, the accumulation of inflammatory cells, red blood cells, and proteinaceous fluid in the alveolar spaces could be easily discovered [30, 31].

### 2.6. Immunohistochemistry

Another portion of the tissue samples was subjected to immunohistochemistry staining to evaluate IL-1 expression [32]. Briefly, 4 *μ*m-thick paraffin-embedded sections were dewaxed twice using xylene and then hydrated in descending grades of ethyl alcohol. Antigen retrieval was performed using a microwave instrument to heat the slides in citrate buffer for 3 minutes. Sections were left to cool down and then treated with 2.5% hydrogen peroxide to block endogenous peroxidase activity. Nonspecific binding was prevented by incubating slides with nonspecific serum for 15 minutes. Slides were covered by the IL-1 antibody (diluted 1 : 100) for 60 minutes and then washed twice with phosphate-buffered saline (PBS). Slides were then covered with the secondary antibody for 30 minutes. Again, slides were washed twice with PBS, and the signal was detected by color development using a DAB chromogen kit (Biocare Medical, USA). Finally, slides were counterstained with Mayer's hematoxylin and mounted with DPX. The primary antibody (IL-1) was omitted in the control slides. Sections were viewed under light microscopy. Analysis of the immunohistochemistry images was performed using Image J software (https://imagej.nih.gov/ij/download.html) according to previously published methods [33].

### 2.7. Statistical Analysis

The ABGs results were expressed as mean ± standard deviation. Data were analyzed using one-way ANOVA followed by the Bonferroni post hoc test. The independent-sample *T*-test was used to compare subjects within each group by sex. A *p*-value of less than or equal to 0.05 was considered statistically significant. Statistical analysis was performed using the SPSS, version 23, statistical software package (IBM Statistics, USA).

## 3. Results

Results were obtained for ABGs, a histological study of rats' lung tissues, immunohistochemical analysis of rats' lung tissues, and an animal well-being assessment based on animal weight and mortality.

### 3.1. Animal Well-Being

**Figure 1:**
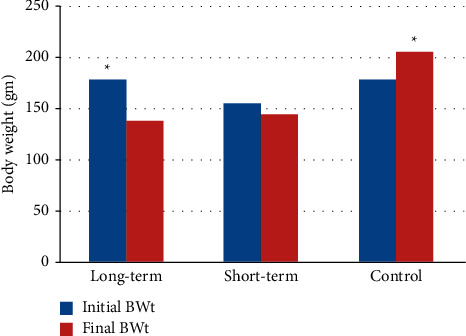
The effects of elemental mercury vapor exposure for the short term (21 days) and long term (65 days) on body weight in rats (*N* = 14).

The exposure of the STG and LTG to 7.3 *μ*l of elemental mercury vapor led to a statistically significant reduction in weight compared with the CG, which gained weight during the experiment. Results are reported in Figure 1. The percentages of body weight loss were 15% and 22% in the STG and LTG, respectively. The CG and STG subjects all survived the course of the study. The LTG had 6 out of the 14 (42.8%) subjects died during the study, and they were two male rats and four females.

### 3.2. Arterial Blood Gas Parameters

Five parameters in the ABGs were compared between groups, and those parameters were pH, PCO_2_, PO_2_, SpO_2_, and HCO_3_. The results for ABGs findings in all groups after short- and long-term exposure to elemental mercury vapor inhalation, as well as the CG, are reported in Table 1. The ABGs testing showed a significant decrease in pH values for the STG and LTG (*p* < 0.05) throughout the study, with no significant change in the control pH values being observed. pH values within the exposed groups were lower than those of the CG (*p* < 0.05). PCO_2_ values were significantly higher in the exposed groups compared with the CG (*p* < 0.001). PO_2_ values were significantly lower in the STG than the CG (*p*=0.03), while no significant difference was found between the LTG and the CG PO_2_ values (*p*=0.21). SpO_2_ values in the STG and LTG were significantly lower than the SpO_2_ in the CG (*p*=0.001). There were no significant differences in the HCO_3_ values between the exposed groups and the CG (*p*=0.506). Also, no significant differences in the examined ABG parameters were found between male and female subjects within the same group in the STG or LTG (*p* > 0.05).

### 3.3. Lung Histopathology

**Figure 2:**
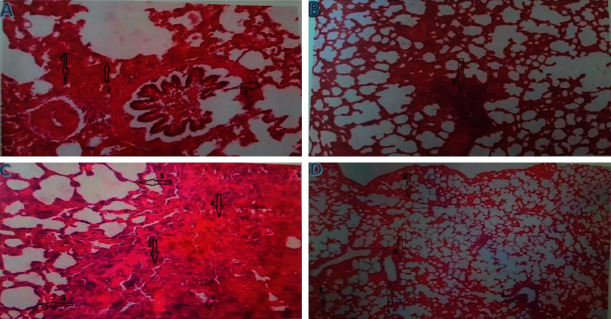
H&E-stained lung tissue sections were obtained from rats after inhalational exposure to mercury vapor. (a) Male with short-term exposure: the section indicates inflammation (arrow 1), emphysema (arrow 2), and alveolar dilatation with the destruction of intra-alveolar septa (arrow 3). (b) Female with short-term exposure: arrow 1 indicates severe inflammation. (c) Male with long-term exposure: the section indicates hypoxia (arrow 1), inflammation (arrow 2), emphysema (arrow 3), and alveolar dilatation with the destruction of intra-alveolar septa (arrow 4). (d) Female with long-term exposure: the section indicates the most severe levels of tissue damage (arrow 1), emphysema (arrow 2), inflammation (arrow 3), hypoxia (arrow 4), and alveolar dilatation with severe destruction of intra-alveolar septa (arrow 5).

Histological study under light microscopy was performed for dissected and prepared lung tissues for all groups. Lung tissue sections obtained from rats after short-term and long-term exposure are presented under light microscopy, as shown in Figure 2. The histological study of lung tissues from the CG revealed mild inflammation. On the other hand, examining lung tissue sections from the STG and the LTG showed marked inflammatory cellular infiltration, emphysema, dilatation of the alveoli intra-alveolar edema, and intra-alveolar septal thickening with destruction and obstruction of intra-alveolar septae. However, the LTG showed a higher degree of lung tissue injury and more severe inflammation than the STG.

### 3.4. Immunohistochemical Study of Rats' Lung Tissues

**Figure 3:**
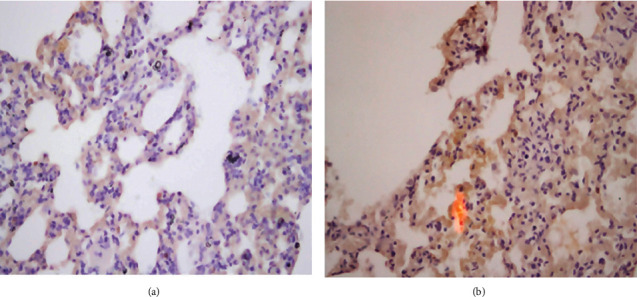
Immunohistochemistry staining to detect IL-1 expression in lung tissues of rats after short-term (a) and long-term (b) exposure to elemental mercury vapor inhalation. No significant differences were observed in IL-1 expression in any of the groups.

Immunohistochemistry staining to detect IL-1 expression in lung tissues was performed and is presented in Figure 3. No signs of significant IL-1 expression were noted in any of the groups.

## 4. Discussion

This study is one of the first scientific studies that evaluated the toxic effects of elementary mercury vapor inhalation on various arterial blood gas parameters in rats and attempted to evaluate possible underlying lung pathology as a direct causal effect of significant alterations in blood acid-base balance. Several side effects related to various body organs and systems have been reported after acute or chronic exposure to elemental mercury [5, 22, 34, 35]. Human exposure to mercury inhalation was reported to cause flu-like symptoms, including fever, cough, dyspnea, and chest pain [36]. In this study, rats in both exposure groups lost weight significantly (*p* ≤ 0.05) during the experiment, while rats in the CG gained weight. A total of 6 out of 14 (43%) rats died, including two males and four females, belonging to the LTG. On the other hand, none of the short-term exposure or control groups' rats died during the experiment. The high mortality rates in the LTG can be explained by severe lung injury, hypoxia, acidemia, and weight loss, observed in such a group, due to the long duration of exposure to elemental mercury vapor.

In both the STG and LTG, there was a significant decrease in blood pH values compared with the CG. This state of severe acidemia was accompanied by a significant increase in blood concentrations of CO_2_ and a substantial reduction in blood concentration and saturation of O_2_ regardless of sex. Simultaneously, no significant changes were observed in blood concentrations of HCO_3_, indicating a lack of renal compensation. However, the PO_2_ and SpO_2_ levels were found to be higher at the end of the study in the LTG than in the STG, indicating some form of adaptive response to the long-term exposure to elemental mercury vapor, leading to improved oxygen saturation of the blood. The ABGs results indicate that rats exposed to elemental vapor inhalation suffered a significant degree of respiratory acidosis, in contrast to previously established evidence in the literature leaning predominantly toward metabolic acidosis with mercury poisoning in both animals [37] and humans, predominantly children [38, 39]. The higher incidence of acidosis in the pediatric age group than in adults exposed to elemental mercury in the literature can be explained by the difference in body weight and physiological maturity, which results in a much lower degree of exposure being necessary to bring mercury to highly toxic and potentially fatal concentrations in the blood and tissues. This carries clinically significant implications in managing pediatric mercury poisoning cases, where healthcare providers should critically consider both metabolic and respiratory acidosis.

Histopathological examination of lung tissues from rats exposed to elementary mercury vapor inhalation revealed substantial pulmonary inflammation characterized by inflammatory cellular infiltration, emphysema, and dilatation of the alveoli with thickening of the intra-alveolar septae. These inflammatory lesions appeared more pronounced in the rats in the LTG than those in the STG. Interestingly, pulmonary lesions were found to be more severe in female rats than male rats. The severe inflammatory lesions of the pulmonary tissues are presumably the underlying cause of the changes noted in the acid-base status of exposed rats. This also is evident by the observed clinical signs of hyperventilation, hypoxia, and weakness. These findings are congruent with previously reported pulmonary lesions in human beings after acute exposure to mercury vapor [40]. Diffuse inflammatory cellular infiltrates, acute pulmonary edema and emphysema, chemical pneumonitis, bronchiolitis, pneumothorax, and even death events were reported in humans after acute mercury vapor inhalation [40]. These findings emphasize the importance of early treatment of lung tissue injury with medications, supplemental oxygen, endotracheal intubation, and mechanical ventilation. These early interventions are crucial to maintaining adequate gas exchange in elemental mercury vapor poisoning to prevent well-known respiratory complications such as hypoxemia and lesser-known significant complications that may arise, such as respiratory acidemia.

In this study, immunohistochemistry staining to detect IL-1 expression in lung tissues revealed no significant differences among all groups. These results disagree with previous findings where mercury vapor inhalation induced increased secretion of IL-1 [19]. The effects of mercury on IL-1 expression have been demonstrated in several laboratory animal models [41]. It has been suggested that exposure of mice to methylmercury induced expression of IL-1 in the brain tissue, causing central nervous cytotoxicity [42]. In contrast, one study found that macrophages from mercury-treated mice exhibited a reduced capacity to produce IL-1 [20]. Therefore, the exact effects of mercury molecules on the production and expression of IL-1 in different body tissues remain controversial, and further clinical trials are warranted to determine the exact pathophysiological impacts of mercury on proinflammatory cytokines production and function.

Despite the novelty of this study in evaluating the toxic effects of elementary mercury vapor inhalation on various arterial blood gas parameters and lung pathology in rates with the presence of a control group and other two groups with different exposure extent, this study has some limitations. First, there is a possibility of selection bias as using portions of the tissue samples for histology and immunohistochemistry evaluations could not provide equal opportunity for analysis and miss the effect of regional heterogeneity. Thus, a sampling scheme should be used that includes all regions to avoid selection bias. However, the evaluated samples were randomly selected. Second, a more detailed evaluation of structural changes in a lung with injury could be achieved using more advanced techniques than a light microscope. The small size of rats places limits on the practical methods used to assess lung injury. However, the light microscope could successfully detect edema and the distribution and nature of structural damage regardless of lung size, but it is still less helpful in evaluating global lung dysfunction [30].

Lastly, the immunohistochemistry staining method was conducted for the detection of IL-1 expression, which is a poorly quantitative technique and has low sensitivity compared with real-time quantitative polymerase chain reaction (Q-PCR) or enzyme-linked immunosorbent assay (ELISA) [43, 44]. However, the immunohistochemistry method has the advantage of detecting small numbers of cytokine-producing cells in a tissue, which might not produce enough cytokine to be detected by the other methods and can localize and insight the distribution of cytokines expression over the cells; thereby the clinical relevance of our results is achievable [43, 44]. Nevertheless, using more than one method for IL-6 and other cytokines detection and measurement in future studies is recommended. For example, both real-time PCR and immunohistochemistry could be used to allow highly quantitative mRNA results using real-time PCR and localization of cytokines producing cells by immunohistochemistry.

## 5. Conclusion

The findings of this study indicate that exposure to elementary mercury vapor induces significant pulmonary injury resulting in severe clinical manifestations, severe respiratory acidosis with hypoxemia, and no evidence of compensatory alkalosis in Sprague Dawley rats. These manifestations are correlated with the duration of mercury vapor exposure regardless of gender. No signs of significant IL-1 expression are noted. Healthcare professionals should consider respiratory acidosis in their treatment plans for patients affected with mercury exposure.

## Figures and Tables

**Table 1:** The arterial blood gas parameters in Sprague Dawley rats after short- and long-term elemental mercury vapor exposure and those with no mercury vapor exposure.

Arterial blood gas parameters	Groups								
	Short-term exposure			Long-term exposure			Control		
	All animals	Males	Females	All animals	Males	Females	All animals	Males	Females
pH	7.23 ± 0.05^*∗*^	7.22 ± 0.03	7.24 ± 0.07	7.18 ± 0.02^*∗*^	7.18 ± 0.02	7.19 ± 0.0	7.36 ± 0.09	7.32 ± 0.02	7.4 ± 0.06
PCO_2_	62 ± 10^*∗*^	65 ± 6	60 ± 13	68 ± 4^*∗*^	69 ± 4	66 ± 4	44 ± 8	50 ± 10	37 ± 4
PO_2_	49 ± 21^*∗*^	42 ± 10	55 ± 18	52 ± 14^*∗*^	55 ± 15	44 ± 5	67 ± 16	57 ± 13	77 ± 11
SpO_2_	66 ± 19^*∗*^	63 ± 17	69 ± 20	71 ± 16^*∗*^	73 ± 18	65 ± 7	89 ± 9	84 ± 10	94 ± 3
HCO_3_	25 ± 3	26 ± 2	24 ± 3	25 ± 2	25 ± 15	25 ± 5	24 ± 3	25 ± 2	23 ± 2

^*∗*^Statistically significant in comparison with the control group (*p* ≤ 0.05).

## Data Availability

The datasets generated and analyzed during the current study are available from the corresponding authors (Liqaa A. Raffee, e-mail: laraffee5@just.edu.jo and Abdel-Hameed Al-Mistarehi, e-mail: awalmistarehi18@med.just.edu.jo) upon reasonable request.
